# Detection of circulating tumour cells before and following adjuvant chemotherapy and long-term prognosis of early breast cancer

**DOI:** 10.1038/s41416-022-01699-5

**Published:** 2022-02-10

**Authors:** Alexios Matikas, Athanasios Kotsakis, Stella Apostolaki, Helen Politaki, Maria Perraki, Kostas Kalbakis, Michalis Nikolaou, Panagiota Economopoulou, Dora Hatzidaki, Vassilis Georgoulias

**Affiliations:** 1 Department of Oncology-Pathology, Karolinska Institutet, Stockholm, Sweden; 2 Breast Center, Theme Cancer, Karolinska University Hospital, Stockholm, Sweden; 3 Department of Medical Oncology, University General Hospital of Larisa, Larisa, Greece; 4 Laboratory of Tumor Cell Biology, University of Crete, Crete, Greece; 5 Department of Medical Oncology, University General Hospital of Heraklion, Crete, Greece; 6 2nd Department of Medical Oncology, “Agios Savas” Anticancer Hospital, Athens, Greece; 7 Medical Oncology Unit, Department of Internal Medicine, “ATTIKON”, University Hospital, Athens, Greece; 8 Hellenic Oncology Research Group, Athens, Greece

**Keywords:** Breast cancer, Tumour biomarkers

## Abstract

**Background:**

The detection of circulating tumour cells (CTC) is prognostic for disease recurrence in early breast cancer (BC). This study aims to investigate whether this prognostic effect persists or varies over time.

**Methods:**

The study population consisted of prospectively included stage I–III BC patients. The presence of *CK19* mRNA-positive CTC in the peripheral blood was evaluated before and after adjuvant chemotherapy, using a real-time RT–PCR assay. Longitudinal samples were collected for a subset of patients.

**Results:**

Baseline CTC data were available from 1220 patients, while 1132 had both pre- and post-therapy data. After a median follow-up of 134.1 months, CTC positivity at baseline was associated with shorter overall survival (OS; HR_adj_ = 1.72, 95% CI 1.34–2.21, *p* < 0.001). For disease-free survival, an interaction with time (*p* = 0.045) was observed. CTC positivity predicted early (within 5 years; HR_adj_ = 1.76, 95_%_ CI 1.33–2.32, *p* < 0.001) but not late recurrence (HR_adj_ = 1.10, 95% CI 0.79–1.53, *p* = 0.577). Following adjuvant chemotherapy, more patients converted from CTC-positive to CTC-negative than vice versa (*p* < 0.001). Ten-year OS was 68.6% for + /+ and 86.7% for −/− group (*p* < 0.001). CTC status at follow-up predicted disease recurrence.

**Conclusion:**

CTC detection pre- and post-adjuvant chemotherapy is prognostic for early relapse, supporting investigations for novel adjuvant therapeutic approaches.

## Introduction

Prognostication for early breast cancer (BC) has traditionally relied on clinic-pathologic factors, such as age [1], tumour size [2], hormone receptor (HR) and human epidermal growth factor receptor 2 (HER2) expression [3, 4], as well as nodal status [2, 5]. Since none of these factors are predictive for relative benefit from adjuvant chemotherapy [6], management decisions are based on the absolute risk for recurrence, with patients at the high-risk spectrum deriving higher absolute benefit from chemotherapy. More recently, gene expression profiling has been shown to provide additional prognostic information to traditional factors and large prospective randomised trials have demonstrated that patients at low genomic risk may not benefit from chemotherapy [7–11].

A relative disadvantage of this combined clinic-genomic approach is that all information is obtained at baseline, without considering how the individual patient responds to adjuvant chemotherapy. In contrast, liquid biomarkers can be obtained both pre- and post-therapy, as well as during the follow-up period, facilitating thus a more individualised prognostication. Baseline enumeration of Circulating Tumour Cells (CTC) in particular has been shown to be strongly prognostic for patient survival [12, 13]. In addition, conversion to CTC-negative from CTC-positive status following chemotherapy distinguishes a population with intermediate prognosis, compared to patients with persistent CTC-negative (best prognosis) or CTC-positive (worst prognosis) status [14]. Whether this prognostic effect persists after a long follow-up period remains unclear. Intriguingly, CTC positivity several years following definite treatment has been associated with late recurrence, albeit with once again short follow-up after CTC detection [15, 16].

Our group has previously demonstrated the prognostic value of CTC based on the detection of *CK19* mRNA in early BC both at baseline [17] and following adjuvant chemotherapy [18], depending on receptor expression [19], the effect of chemotherapy [20] and endocrine therapy [21] on CTC status, and the feasibility of serial measurements for prognostication [22]. However, published studies at the adjuvant setting have commonly been underpowered and with inadequate follow-up in order to assess whether this prognostic effect persists or varies through time [12], which the present study aimed to address.

## Methods

### Description of the patient cohort

This is a retrospective analysis of prospectively collected data. A total of 1220 women with operable early or locally advanced, non-metastatic (stage I–III) BC that were treated from 1997 to 2019 in two hospitals in Greece (University Hospital of Heraklion, Crete and Metropolitan General Hospital, Athens) were included in this study. Most of these patients were treated within the context of prospective randomised trials conducted by the Hellenic Oncology Research Group regarding various adjuvant chemotherapy regimens, which have been previously reported [23–26]. Patients not treated within a prospective clinical trial received postoperative therapy in accordance with local practice and national guidelines.

Patient follow-up consisted of medical history and physical examination, with laboratory and imaging studies as indicated, every 3 months for the first 2 years, every 6 months for the next 3 years and yearly thereafter. All treating physicians were blinded to the CK19 mRNA results for their individual patients and all follow-up laboratory and imaging studies to detect disease relapse were performed independently of the CK19 mRNA results. All patients gave their written informed consent for the assessment of CTC status and all prospective clinical trials, as well this specific study on CTC detection, were approved by the Ethics and Scientific Committees of the two institutions.

### Methodology of detection of *CK19* mRNA-positive CTC

Peripheral blood (20 ml in EDTA) was collected for each sample. To avoid contamination with epithelial skin cells, all blood samples were obtained at the middle of vein puncture after the first 5 mL of blood was discarded. Sample collection occurred 3–4 weeks after primary surgery and before the initiation of adjuvant chemotherapy, as well as 3–4 weeks after the completion of adjuvant chemotherapy as previously reported [21, 22, 27]. In addition, longitudinal samples were collected for a subset of patients, every 6 months for the first five years following radical surgery and then annually (Supplementary Fig. 1). Persistent CTC positivity was defined as positive status both pre- and post-therapy, at the time the specific sample was obtained.

The procedures of RNA extraction and cDNA synthesis, the real-time RT–PCR assay for the detection of *CK19* mRNA-positive CTC as well as its specificity and sensitivity have been previously reported in detail [27]. According to the analytical detection limit of the assay, the presence of ≥0.6 MCF-7 equivalents/5 μg of total RNA was considered as a positive result, as previously described in detail [22, 27].

### Statistical analysis

The aim of this study was to explore whether the prognostic value of CTCs based on the detection of *CK19* mRNA persists or varies over time. Due to its descriptive nature, no formal statistical hypothesis was tested. Summary tables (descriptive statistics and/or frequency tables) were provided for all baseline and efficacy variables, as appropriate. Continuous variables were summarised with descriptive statistics (n, median and range). Qualitative factors were compared by Pearson’s chi-squared test or Fisher’s exact test whenever appropriate. Differences in positivity rates were assessed using the McNemar test while differences in continuous variables were assessed using the Mann–Whitney U test. Disease-free survival (DFS) was defined as the time from the start date of the treatment to the date of BC recurrence (either locoregional or distant), contralateral BC diagnosis, non-breast second primary cancer or death from any cause, whichever occurred first. Distant relapse-free survival (DRFS) was defined as the time from the start date of the treatment to the date of distant relapse or death from any cause. Overall survival (OS) was defined as the time from the start date of the treatment to the date of death from any cause. Patients alive without any predefined event (relapse or death) were censored at the time of the last known assessment. Time-to-endpoint events (DFS, DRFS, OS) were estimated using the Kaplan–Meier method and the comparisons were computed with the log-rank test. Median follow-up was calculated using the reverse Kaplan–Meier method. The independent effect of presence or absence of CTC at baseline on DFS and OS was examined by fitting a Cox proportional hazards regression model including other potential prognostic factors in the model (age, menopausal status, tumour size, nodal status, tumour grade, hormone receptors, HER2) with the computation of hazard ratios (HRs) and 95% confidence intervals. The interaction between CTC status and breast cancer subtype with respect to survival outcomes was evaluated in regression models. All statistical tests were two-sided and *p*-values <0.05 were considered statistically significant and were not adjusted for multiple comparisons. Data were analysed using the SPSS statistical software, version 22.0 (SPSS Inc., Chicago, IL, USA).

## Results

### Patient characteristics

In total, 1220 patients had available CTC data at baseline (Supplementary Fig. 1). The patients’ demographic, clinical and pathologic characteristics, as well as their distribution according to CTC status (positive versus negative), are presented in Table 1. Patients with detectable *CK19* mRNA CTC at baseline (*n* = 483; 39.6%) had larger tumours (Pearson’s chi-square *p* = 0.010), more often positive nodal status (Pearson’s chi-square *p* = 0.028) and higher expression of Ki-67 (Pearson’s chi-square *p* = 0.001). Receptor expression and distribution of immunohistochemistry-based subtypes did not differ between CTC-positive and CTC-negative patients. The majority (*n* = 1177 patients, 96.5%) received adjuvant chemotherapy, most commonly anthracycline- and taxane-based combinations (61.2%), followed by anthracycline-based regimens (20.3%) and docetaxel/cyclophosphamide (9.0%).

**Table 1:** Patients’ clinicopathologic characteristics in entire cohort and per baseline CTC status.

	All Pts (*n* = 1220)	CTCs (+) (*n* = 483)	CTCs (−) (*n* = 737)	
	*N* (%)	*N* (%)	*N* (%)	*P*-value
Age				
Median (min–max)	53 (25–83)	54 (26–83)	53 (25–81)	0.870
Menopausal status				
Pre-menopausal	552 (45.2)	225 (46.6)	327 (44.4)	0.447
Post-menopausal	668 (54.8)	258 (53.4)	410 (55.6)	
Tumour size				
≤2 cm	505 (45.1)	179 (37.1)	326 (44.5)	0.010
>2 cm	710 (63.4)	304 (62.9)	406 (55.5)	
NA	5 (0.4)			
Nodal status				
Negative	460 (37.7)	164 (34.2)	296 (40.4)	0.028
Positive	752 (61.6)	316 (65.8)	436 (59.6)	
NA	8 (0.7)			
Oestrogen receptor				
Positive	809 (66.3)	319 (66.5)	490 (67.3)	0.759
Negative	399 (32.7)	161 (33.5)	238 (32.7)	
NA	12 (1.0)			
Progesterone receptor				
Positive	757 (62.0)	291 (60.6)	466 (64.2)	0.210
Negative	449 (36.8)	189 (39.4)	260 (35.9)	
NA	14 (1.2)			
HER2				
Positive	247 (20.2)	93 (19.5)	154 (21.3)	0.468
Negative	953 (78.1)	383 (80.5)	570 (78.7)	
NA	20 (1.6)			
Subtype				
ER+/HER2−	755 (61.9)	291 (66.3)	464 (64.3)	0.100
HER2+	244 (20.0)	92 (19.4)	152 (21.1)	
TNBC	198 (16.2)	92 (19.4)	106 (14.7)	
NA	23 (1.9)			
Grade				
I	24 (2.2)	6 (1.4)	18 (2.7)	0.247
II	541 (48.9)	223 (50.7)	318 (47.7)	
III	541 (48.9)	211 (48.0)	330 (49.5)	
Ki-67				
≤20%	336 (27.5)	107 (48.9)	229 (63.1)	0.001
** >**21%	246 (20.2)	112 (51.1)	134 (36.9)	
NA	638 (52.3)			
Type of surgery				
BCS	810 (66.4)	314 (65.0)	496 (67.3)	0.179
Mastectomy	406 (33.3)	169 (35.0)	237 (32.2)	
NA	4 (0.3)	-	4 (0.5)	
Adjuvant chemotherapy				
TC	110 (9.0)	31 (6.4)	79 (10.7)	0.066
Anthracycline	248 (20.3)	105 (21.7)	143 (19.4)	
Anthracycline and Taxane	747 (61.2)	308 (63.8)	439 (59.6)	
Other Chemo	72 (5.9)	27 (5.6)	45 (6.1)	
Endocrine only	38 (3.1)	11 (2.3)	27 (3.7)	
No adjuvant	5 (0.4)	1 (0.2)	4 (0.5)	
Adjuvant endocrine				
Yes	976 (80.0)	375 (77.6)	601 (81.5)	0.095
No	244 (20.0)	108 (22.4)	136 (18.5)	

*CTC* circulating tumour cell, *ER* oestrogen receptor, *HER2* human epidermal growth factor receptor 2, *TC* docetaxel/cyclophosphamide, *TNBC* triple-negative breast cancer.

### Baseline CTC status and prognostication

**Fig. 1:**
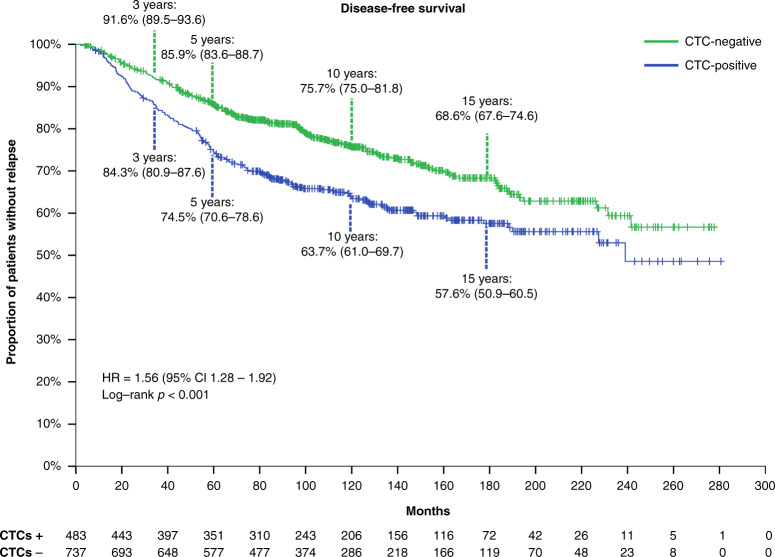
Kaplan–Meier curve for disease-free survival according to baseline (pre-therapy) CTC status. CI confidence interval, CTC circulating tumour cells, HR hazard ratio.

After a median follow-up of 134.1 months (range, 3.9–280.6 months), 372 patients (30.5%) had relapsed, 186 (38.5%) from the CTC-positive and 186 (25.2%) from the CTC-negative group. The majority (65.0%) of DFS events were distant metastases (Supplementary Tables 1 and 2). The corresponding 10-year DFS probability was 63.7% (95% Confidence Interval [CI] 61.0–69.7%) for CTC-positive versus 75.7% (95% CI 75.0–81.8%) for CTC-negative patients (*p* < 0.001, Fig. 1).

**Fig. 2:**
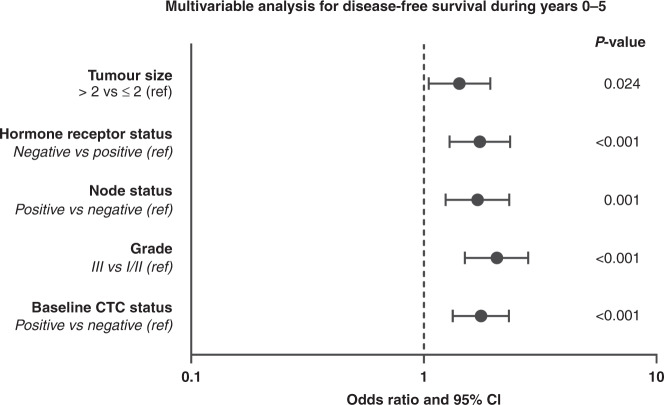
Multivariable Cox regression model for the endpoint of disease-free survival during years 0–5. CI confidence interval, CTC circulating tumour cell, ref reference.

An interaction between baseline CTC status and time was observed for DFS (*p* = 0.045). When looking into patients with early relapse during the first 5 years following diagnosis, baseline CTC positivity was independently prognostic for shorter DFS at the multivariable analysis when adjusting for age, tumour size, nodal status, grade and hormone receptor status (HR_adj_ = 1.76, 95% CI 1.33–2.32, *p* < 0.001; Fig. 2). This association was observed across IHC subtypes (p_interaction_ 0.173), although wide confidence intervals were noted for the HER2 + subgroup due to the low number of events (Supplementary Table 3). In contrast, baseline CTC status was not prognostic for delayed (>5 years) relapse (HR = 1.10, 95% CI 0.79–1.53, *p* = 0.577). Similar results were noted for the DRFS endpoint, with baseline CTC positivity being associated with worse DRFS during years 0–5 (HR_adj_ = 1.90, 95% CI 1.42–2.56, *p* < 0.001) but not beyond 5 years (HR_adj_ = 1.23, 95% CI 0.87–1.76, *p* = 0.243). The lack of prognostic value for delayed relapse was apparent even when focusing on HR-positive disease (univariate Cox regression HR = 1.31, 95% CI 0.89–1.92, *p* = 0.169).

Similarly, 284 (23.3%) patients died during the follow-up period, 153 (31.7%) from the CTC-positive and 131 (17.8%) from the CTC-negative group for a 10-year overall survival rate of 72.4% (95% CI 70.6–78.6%) versus 85.2% (95% CI 85.0–90.0%), respectively (*p* < 0.001). Baseline CTC positivity remained an independent negative prognostic factor at multivariable analysis (HR_adj_ = 1.72, 95% CI 1.34–2.21, *p* < 0.001) as shown in Table 2 and Supplementary Table 4.

**Table 2:** Univariate and multivariable analysis of baseline clinicopathologic factors for overall survival.

	Univariate analysis		Multivariable analysis	
Factors	Hazard ratio (95% C.I)	*P*-value	Hazard ratio (95% C.I)	*P*-value
Age		<0.001		0.015
≤60	1 (reference)		1 (reference)	
>60	1.64 (1.29–2.08)		1.38 (1.07–1.78)	
T size		<0.001		<0.001
≤2 cm	1 (reference)		1 (reference)	
>2 cm	2.38 (1.81–3.13)		1.89 (1.41–2.53)	
Nodes		<0.001		<0.001
Negative	1 (reference)		1 (reference)	
Positive	1.95 (1.39–2.29)		1.85 (1.37–2.50)	
Grade		<0.001		0.002
I/II	1 (reference)		1 (reference)	
III	1.78 (1.39–2.29)		1.54 (1.18–2.02)	
Hormone receptor		0.022		0.022
Positive	1 (reference)		1 (reference)	
Negative	1.35 (1.04–1.74)		1.39 (1.05–1.84)	
CTC		<0.001		<0.001
Positive	1.74 (1.38–2.20)		1.72 (1.34–2.21)	
Negative	1 (reference)		1 (reference)	

*CI* confidence interval, *CTC* circulating tumour cell.

### Post-therapy CTC dynamics and prognostication

Both baseline and immediately post-therapy CTC status were available from 1132 patients (92.7% of the entire cohort). *CK19* mRNA was detected in both samples (+/+) in 225 patients (19.9%); 229 patients (20.2%) had positive pre-therapy but negative post-therapy CTC status (+/−); 114 patients (10.1%) had the opposite (−/+), while 564 patients (49.8%) were persistently CTC-negative. More patients converted from CTC-positive to CTC-negative following adjuvant chemotherapy, than vice versa (McNemar’s test *p* < 0.001). This pattern was noted across IHC subtypes (Supplementary Table 5).

**Fig. 3:**
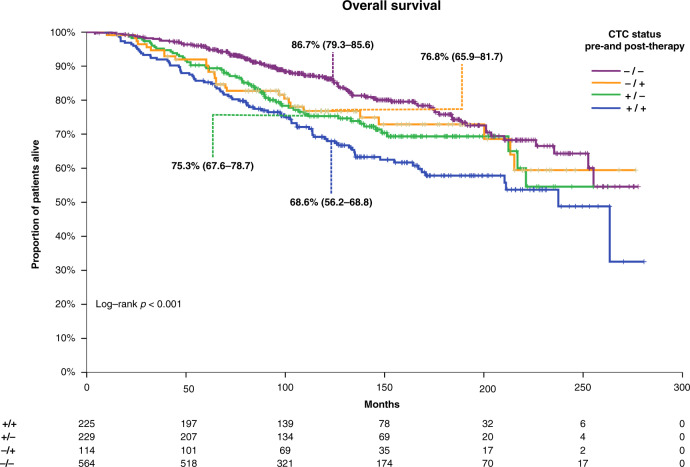
Kaplan–Meier curve for overall survival according to pre- and post-therapy CTC status. CTC circulating tumour cells.

Long-term survival outcomes differed between these groups: 10-year DFS rates were 61.1% (95% CI 55.7–68.3%) for +/+ group, 67.1% (95% CI 62.7–74.6%) for +/− group, 65.2% (95% CI 59.4–76.2%) for −/+ group and 78.1% (95% CI 75.4–82.1%) for −/− group (log-rank test: *p* < 0.001). The corresponding 10-year OS rates are shown in Fig. 3.

Compared with patients in the −/− group, those in the +/+ had worse DFS and OS in multivariable analysis when adjusted for age, size, nodal status, grade and receptor status (DFS: HR_adj_ = 1.88, 95% CI 1.42–2.49, *p* < 0.001; OS: HR_adj_ = 2.12, 95% CI 1.54–2.91, *p* < 0.001). Similar results were noted for patients in the +/− and −/+ compared with the −/− group [(DFS: HR_adj_ = 1.61, 95% CI 1.20–2.18, *p* = 0.002; OS: HR_adj_ = 1.81, 95% CI 1.28–2.57, *p* = 0.001) and (DFS: HR_adj_ = 2.09, 95% CI 1.48–2.97, *p* < 0.001; OS: HR_adj_ = 1.90, 95% CI 1.23–2.91, *p* = 0.003)], respectively. However, conversion of CTC status from baseline positive to post-therapy negative did not improve prognosis both in terms of DFS (HR = 0.79, 95% CI 0.59–1.07, *p* = 0.126) and OS (HR = 0.74, 95% CI 0.53–1.03, *p* = 0.072), compared to patients that were persistently positive.

Compared with patients in the −/− group, patients with detection of CTC pre- and post-therapy had worse short-term (years 0–5) DFS (HR = 2.09, 95% CI 1.45–3.0, *p* < 0.001). However, persistent CTC positivity was not prognostic for late relapse after the 5-year timepoint (HR = 1.28, 95% CI 0.83–1.97, *p* = 0.266). Similar results were noted for the DRFS endpoint, for both short-term (HR_adj_ = 2.33, 95% CI 1.59–3.42, *p* < 0.001) and long-term prognosis (HR_adj_ = 1.34, 95% CI 0.85–2.11, *p* = 0.214).

### CTC status during follow-up and risk for delayed relapse

Serial CTC samples during follow-up were obtained from a subgroup of 185 patients. Their baseline characteristics are presented in Supplementary Table 6, while data availability is shown in Supplementary Fig. 1 and Supplementary Table 7. Data from four timepoints at 1, 2, 3 and 5 years pos-toperatively are presented hereunder, with 62 (33.5%) patients having serial samples from all four timepoints, and 157 (84.9%) having from at least two. Depending on the timepoint, up to 19.8% of patients had detectable CTC (Supplementary Table 8). Of the 97 patients with -/- status pre- and post-therapy that had available longitudinal data at least once during follow-up, 8.9% were positive at 12 months (8/90; Fisher’s exact test *p* < 0.001), 5.2% at 24 months (4/77; Fisher’s exact test *p* < 0.001), 10% at 36 months (6/60; Fisher’s exact test *p* = 0.030) and 9.8% at 60 months (4/41; Fisher’s exact test *p* < 0.001).

CTC positivity at these four timepoints was generally associated with worse outcomes, although wide confidence intervals were noted due to the low number of events (at 12 months: HR = 2.36, 95% CI 1.15–4.85, *p* = 0.02; at 24 months: HR = 5.72, 95% CI 2.38–13.78, *p* < 0.001; at 36 months: HR = 1.92, 95% CI 0.66–5.6, *p* = 0.229; at 60 months: HR = 3.01, 95% CI 0.62–14.52, *p* = 0.169).

## Discussion

In this large retrospective analysis of samples collected from prospectively enrolled patients with a median follow-up of more than 11 years, we demonstrate that the prognostic effect of both pre- and post-therapy CTC status varies through time, with CTC detection being prognostic for early disease recurrence. In addition, obtaining new CTC samples during follow-up could provide additional prognostic information for patients already surviving without relapse several years following primary surgery. Despite the exploratory nature of our study, these two findings have potential clinical implications, since they support the role of CTC for risk stratification that might affect management decisions at the adjuvant setting. On one hand, by supporting novel individualised adjuvant strategies aiming to eradicate the minimal residual disease (MRD) and on the other hand, by distinguishing a population with MRD years following primary treatment and thus potentially in need of extended endocrine therapy.

An inherent disadvantage of adjuvant chemotherapy is that there is no indication whether the individual patient has already been cured, leading potentially to both over- and under-treatment. Prolonging the duration of adjuvant chemotherapy indiscriminately does not improve outcomes [28]. Monitoring MRD following surgery and chemotherapy, either with CTC, circulating tumour DNA or extracellular vesicles, is an avenue worth pursuing in an effort to individualise further treatment [29]. Novel adjuvant approaches aiming to eradicate MRD are emerging following the highly successful paradigm of prolonged HER2-blockade, such as long-term metronomic adjuvant chemotherapy for triple-negative BC [30] or adjuvant cyclin-dependent kinase (CDK) 4/6 inhibitors for HR-positive, HER2-negative BC [31]. The conflicting results regarding the latter underscore the need for better risk stratification and patient selection [32, 33]. Baseline and post-therapy CTC positivity was associated with worse outcomes in our study, with detectable MRD being an independent predictor for early relapse. Although speculative, this patient population may be an excellent target group for early consolidation therapy. Whether this approach is successful in eradicating MRD and improving survival should be the aim of prospective randomised studies with the endpoint of CTC clearance. Small randomised studies of HER2 targeted agents for HER2-negative BC have demonstrated the feasibility of this approach and reported promising results [34–36].

The natural history of early BC, especially HR-positive disease, is prolonged with a steady annual rate of recurrence and a continuously increasing cumulative risk of recurrence [37]. While extended endocrine therapy has been shown to marginally decrease the risk for recurrence [38], patient selection is challenging. Clinical and pathologic factors [39] and gene expression profiling [40–43] are used to select patients at high risk for late recurrence. However, such an approach is based on baseline risk stratification and ignores the fact that many patients with HR-positive disease surviving at least 5 years are presumably cured and thus overtreated if exposed to extended therapy [44]. As a result, the selection of patients for extended endocrine therapy according to persistent MRD is an intriguing approach. In our study, persistent CTC positivity at 1, 2, 3 and 5 years following adjuvant chemotherapy was associated with worse patient outcomes, confirming previous reports on CTC enumeration based on another assay [15, 16]. Further studies are needed to show if this adverse outcome may be overcome by continuing or changing endocrine therapy.

Despite the large size and prolonged follow-up of our study, several limitations of our exploratory analysis should be acknowledged. Firstly, various chemotherapy regimens were used, and patient allocation to treatment was not randomised. As a result, the relative contribution of chemotherapy to CTC status conversion and prognosis cannot be assessed. Furthermore, robust conclusions regarding the potential impact of CTC clearance cannot be drawn. While CTC persistence post-therapy is a potential marker of chemoresistance, their disappearance is a function of the sensitivity of the method, thus multiple longitudinal samples are needed for confirmation. However, in our study there were few patients with CTC samples at long-term follow-up and few events in this group, rendering these analyses purely exploratory and prone to associations by chance, considering that the increase in the familywise error rate was not controlled in the reported analyses. Finally, the methodology used for CTC detection in this study is developed in-house and is not approved for routine clinical use. Nevertheless, the overall agreement of this assay with the more commonly used CellSearch system (Menarini Silicon Biosystems Inc, Italy) is up to 73.5%, depending on the CTC cut-off [45].

In conclusion, the prognostic effect of pre- and post- adjuvant chemotherapy CTC status persists after long follow-up. Detection of baseline and post-therapy MRD was shown to be associated with early and not delayed relapse, which supports further investigations for alternative adjuvant therapy approaches. The potential clinical implications support the conduct of randomised studies of early BC with CTC clearance as the primary endpoint.

## Supplementary information

Supplementary Material

Checklist

## Data Availability

The datasets that support the findings of this study are available from the corresponding (AM) or the senior author (VG) upon reasonable request.
